# The differential expression pattern of the *BMI-1, SALL4* and *ABCA3* genes in myeloid leukemia

**DOI:** 10.1186/1475-2867-12-42

**Published:** 2012-10-15

**Authors:** Qi Shen, Sichu Liu, Junyan Hu, Shaohua Chen, Lijian Yang, Bo Li, Xiuli Wu, Yu Ma, Jianchang Yang, Yupo Ma, Yangqiu Li

**Affiliations:** 1 Institute of Hematology, Jinan University, Guangzhou, 510632, China; 2 Department of Surgery,BST-9, School of Medicine, Stony Brook University, Stony Brook, NY, 11794, USA; 3 Department of Pathology, BST-9, School of Medicine, Stony Brook University, Stony Brook, NY, 11794, USA; 4 Key Laboratory for Regenerative Medicine of Ministry of Education, Jinan University, Guangzhou, 510632, China

**Keywords:** *SALL4* gene, *BMI-1* gene, Real-time PCR, AML, CML

## Abstract

**Background and methods:**

In order to characterize the expression pattern of *SALL4*, *BMI-1* and ABCA3 genes in patients with myeloid leukemia and those who achieved complete remission (CR) after chemotherapy. Real-time PCR was used to determine the expression level of these genes in peripheral blood mononuclear cells from 24 patients with AML, eight patients with AML-CR, 13 patients with CML in the chronic phase (CML-CP), 12 patients with CML in blast crisis (CML-BC), 13 patients with CML-CR and 11 healthy individuals (HI).

**Results:**

Overexpression of the *BMI-1* gene was found in the AML, CML-CP and CML-BC groups as compared with HI group, while the BMI-1 expression level was lower in patients who achieved CR. In contrast, significantly increased *SALL4* expression was only found in AML group, additionally, *SALL4* expression was lower in the CML-CP and CML-CR groups compared with the HI group, while the *SALL4* expression level in the CML-BC group was higher and significantly greater than that in the CML-CP and CML-CR groups. Moreover, a positive correlation between the expression of *SALL4 and BMI-1* genes was found in samples from most groups. There was no significant difference of *ABCA3* expression level in AML and CML-BC group in comparison with HI group. Interestingly, the *ABCA3* expression level was significantly decreased in the CML-CP, AML-CR and CML-CR in comparison with the HI group. Moreover, the *ABCA3* expression level in all of the CR groups was lower than that in their corresponding groups.

**Conclusions:**

These results describe the altered *SALL4, ABCA3* and *BMI-1* expression pattern in different phases of myeloid leukemia, which may relate to the development and progression to different diseases. *SALL4* expression was strongly correlated with *BMI-1* in most of the myeloid leukemia patient groups, providing a potential link between *SALL4* and *BMI-1* in leukemogenesis.

## Background

The altered expression of genes, such as *WT1*, *SCL*, and *Notch1,* that play crucial roles in the regulation of hematopoietic progenitor cell proliferation is frequently found in leukemia [1-7]. Increasing data show that the genes involved in hematopoietic stem/progenitor cell (HSPC) proliferation change their expression pattern during leukemogenesis [8].

*SALL4* (sal-like protein 4), a *SALL* gene family member that is a newly identified zinc-finger transcription factor, was originally cloned based on its sequence homology to *Drosophila spalt* (*sal*) [9-12]. Alternative splicing generates two variant forms of human *SALL4* mRNA, *SALL4A* and *SALL4B*, and each has a different tissue distribution [9,13]. Recently, *SALL4* has been shown to play an important role in maintaining ES cell (ESC) pluripotency and self-renewal properties. *SALL4* is involved in the self-renewal of leukemic initiation and HSPC [14]. Moreover, recent data have shown that *SALL4* plays an essential role in myeloid leukemogenesis. *SALL4* is constitutively expressed in human leukemia cell lines and primary acute myeloid leukemia (AML) cells [9,13]. Transgenic mice that ubiquitously overexpress *SALL4B* exhibit myelodysplastic syndrome (MDS)-like symptoms and subsequently develop transplantable AML [9,13], while *SALL4* knockdown in leukemia cell lines triggers apoptosis [15].

BMI-1 is a member of the polycomb group of proteins, and it was initially identified in *Drosophila* as a repressor of homeotic genes [9,16-18]. The *BMI-1* gene was initially isolated as an oncogene that cooperates with c-myc in retroviral-induced B and T cell leukemia [19,20]. In humans, *BMI-1* is highly expressed in purified HSCs, and its expression declines with differentiation [9,21], and it plays an essential role in regulating adult, self-renewing HSPC and leukemia stem cells [9,21-27]. Knockout of the *BMI-1* gene in mice results in the progressive loss of all hematopoietic lineages [9,25]. *BMI-1* expression appears to be important for the accumulation of leukemic cells. Interestingly, inhibiting tumor stem cell self renewal after *BMI-1* deletion can prevent leukemic recurrence. Recently, *BMI-1* expression has been used as an important marker for predicting MDS development and the progression to AML [9,28]. *BMI-1* overexpression was also observed in a significant number of nasopharyngeal carcinoma tumors that correlated with advanced tumor progression, invasive stage and poor prognosis [19,29].

*BMI-1* was recently demonstrated to be a direct *SALL4* target gene. The induction of *SALL4* expression is associated with increased levels of histone H3–K4 and H3–K79 methylation in the *BMI-1* promoter, indicating a novel connection between *SALL4* and polycomb group proteins in leukemogenesis and a mechanism whereby aberrant *SALL4* expression can directly alter BMI-1 expression [9].

Moreover, *SALL4* expression was higher in drug resistant primary acute myeloid leukemic patients than those from drug-responsive cases. In addition, *SALL4* expression was enriched in the SP when compared to the non-SP counterpart. Recently, it is reported that *SALL4* could promote the expression of the ABC transporter genes, such as ATP binding cassette transporter A3 (*ABCA3*), suggesting that *SALL4* can contribute to the SP phenotype by regulating the expression of *ABCA3* and *ABCG2*[15].

*ABCA3* is a member of the ATP-binding cassette (ABC) family of transport proteins and is required for perinatal respiratory adaptation. Mutations in *ABCA3* resulted in fatal neonatal lung disease [30,31]. *ABCA3* is highly expressed in AML and ALL patient samples and its expression is associated with unfavorable clinical treatment outcome. Furthermore, the expression of *ABCA3* is enriched in leukemic SP cells and has been linked to multidrug resistance by facilitating lysosomal sequestration of drugs in AML primary cells and cell lines [15,32-35]. RNAi specific for *ABCA3* led to a decrease of *ABCA3* expression in T-ALL cell line such as CCRF-CEM and Jurkat cells. Consequently, a significant sensitization of cells to cytostatic drugs was achieved [35]. Moreover, both pharmacological blockade and the silencing of *ABCA3* enhanced susceptibility of target B-cell lymphoma cells to anti-CD20 antibody-mediated lysis. Mechanisms of cancer cell resistance to drugs and antibodies are linked in an *ABCA3*-dependent pathway of exosome secretion [36].

Little is known about the expression pattern of the *SALL4, ABCA3* and *BMI-1* genes in patients with myeloid leukemia and patients that achieved complete remission after chemotherapy. In this study, we determined the expression characteristics of the *SALL4, ABCA3* and *BMI-1* genes in de novo AML and CML and complete remission samples.

## Results

The high amplification efficiency of the *BMI-1* and *SALL4* genes was consistent with that of the *β2M* reference gene. The PCR products from all of the genes of interest were confirmed using 2.5% agarose gel electrophoresis followed by sequencing (data not shown). The *BMI-1* and *SALL4* genes were detected in all of the PBMC samples from the healthy individuals and those with myeloid leukemia.

### Higher expression of *BMI-1* in AML and CML

*BMI-1* overexpression was found in the de novo AML (median: 0.303, p <0.001), CML-CP (median: 0.295, p=0.006), and CML-BC (median: 0.109, p=0.01) groups in comparison with the HI group (median: 0.027); the *BMI-1* expression level in the AML-CR group (median: 0.078) was not significantly different compared with the HI group (p=0.322). Interestingly, the *BMI-1* expression level in the CML-CR group (median: 0.003) was significantly lower than that in the HI (p< 0.0001) and CML groups (p< 0.0001).

**Figure 1:**
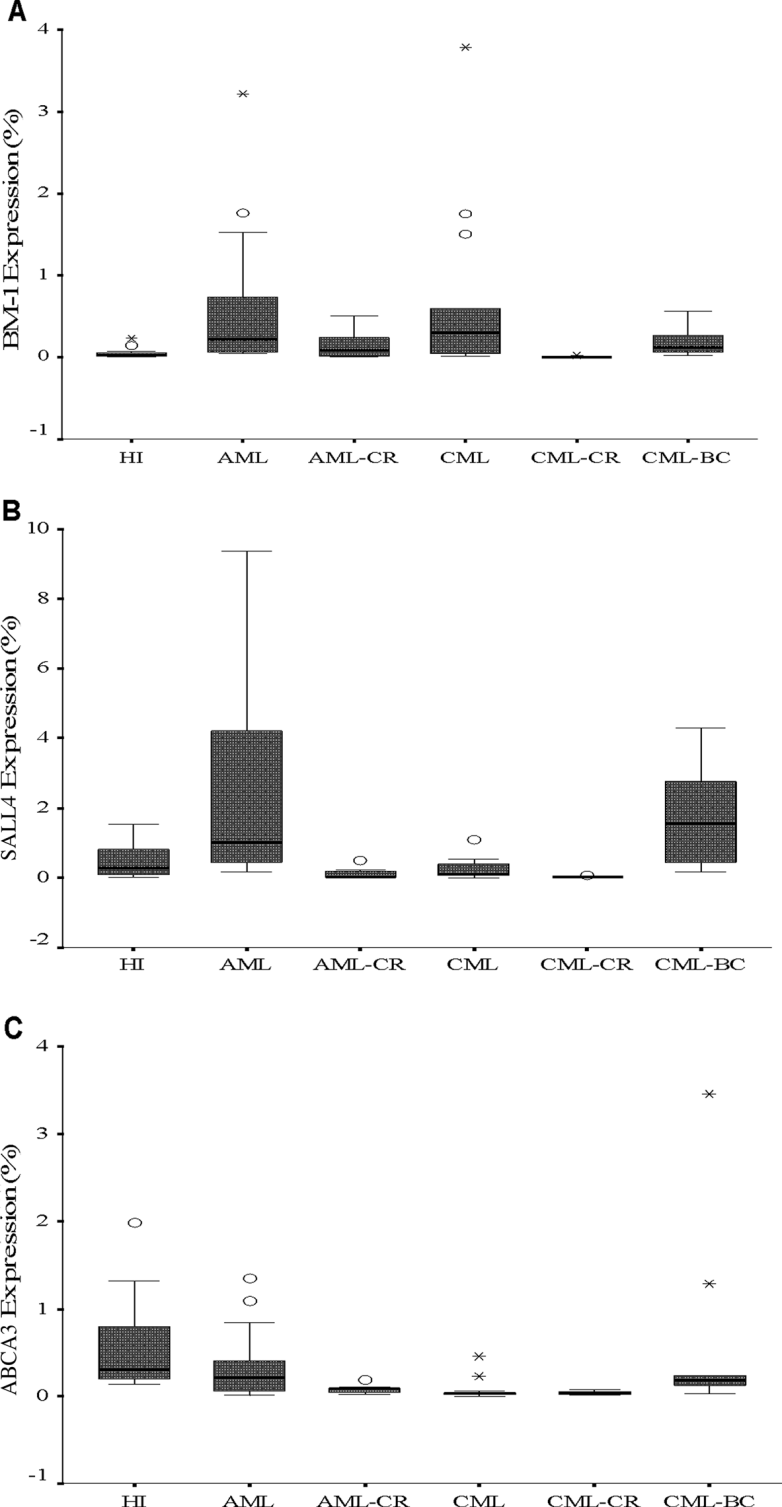
**The *BMI-1 *(A), *SALL4 *(B) and *ABCA3 *(C) expression levels in the different myeloid leukemia groups and healthy individuals.**

**Figure 2:**
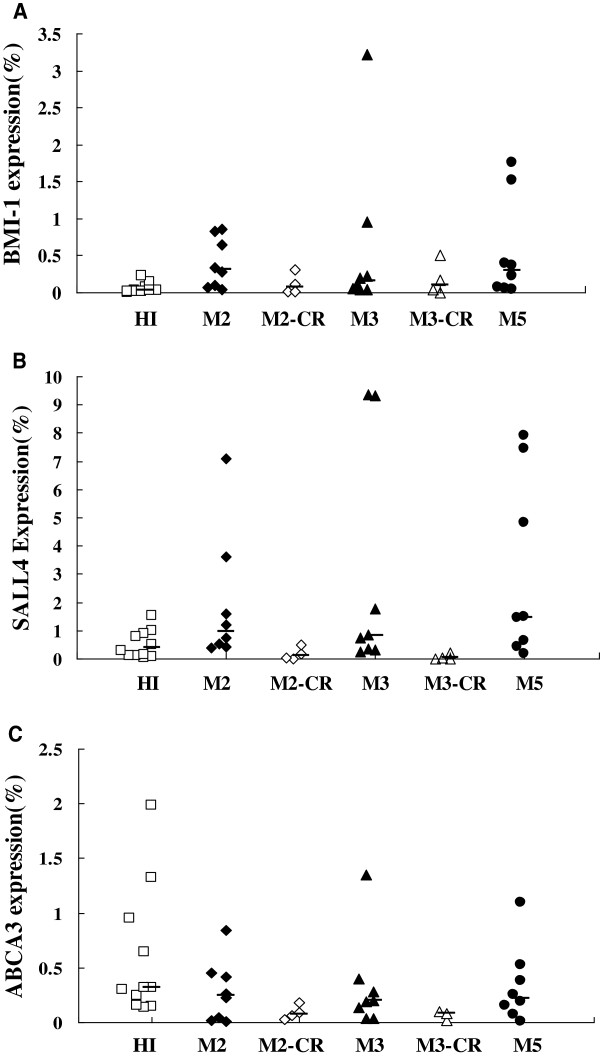
**The *BMI-1 *(A), *SALL4 *(B) and *ABCA3 *(C) expression levels in the different AML subtypes and healthy individuals.**

We next analyzed *BMI-1* expression in different AML subtypes, including M2 (median: 0.400), M3 (median: 0.156) and M5 (median: 0.295), and all had a significantly higher expression level compared to the HI group (p=0.003, p=0.01 and p=0.004, respectively), while the *BMI-1* expression level in the M2-CR (median: 0.070) and M3-CR (median: 0.099) groups was not significantly different in comparison to that of the HI group (p=0.514 and p=0.361, respectively) (Figures 1 and 2).

### Different expression pattern of *SALL4* in AML and CML

The expression pattern appeared different for *SALL4* in the different myeloid leukemia in comparison with the HI group (median: 0.394). While the *SALL4* expression level was increased in the AML group (median: 1.051; p=0.009), CML-BC group (median: 1.563; p=0.016) and the different AML subtypes M2 (median: 0.974; p=0.039), M3 (median: 0.799; p=0.083) and M5 (median: 1.465; p=0.026), it was lower in the AML-CR (median: 0.026; p=0.026), M2-CR (median: 0.105; p=0.151) and M3-CR (median: 0.023; p=0.037) groups. Interestingly, the level of *SALL4* expression in the CML-CP (median: 0.093; p=0.213) and CML-CR groups (median: 0.025; p<0.0001) was lower in comparison with the HI group, and the increased *SALL4* expression level in the CML-BC group (median: 1.563) was significantly higher than that in the CML-CP (p=0.001) and CML-CR (p<0.0001) groups. The *SALL4* expression level in all of the CR groups was lower than that in their corresponding groups i.e., AML vs. AML-CR (p< 0.0001), M2 vs. M2-CR (p=0.017), M3 vs. M3-CR (p=0.007) and CML vs. CML-CR (p=0.011) (Figures 1 and 2).

### Low expression of *ABCA3* in myeloid leukemia

The expression level of *ABCA3* seemed low in the different myeloid leukemia in comparison with the HI group. There was no significant difference of *ABCA3* expression level in AML (median: 0.211; p=0.136), CML-BC (median: 0.174; p=0.097) and the different AML subtypes M2 (median: 0.242; p=0.215), M3 (median: 0.195; p = 0.186) and M5 (median: 0.221; p=0.364) in comparison with the HI group (median: 0.313). While the *ABCA3* expression level was significantly decreased in the CML-CP (median: 0.025; p <0.0001), AML-CR (median: 0.078; p=0.0011) and CML-CR (median: 0.037; p <0.0001) in comparison with the HI group. Moreover, the *ABCA3* expression level in all of the CR groups was lower than that in their corresponding groups i.e., AML vs. AML-CR (p=0.042) (Figures 1 and 2).

### Correlation of relative expression of *BMI-1*, *SALL4* and *ABCA3* in myeloid leukemia

**Figure 3:**
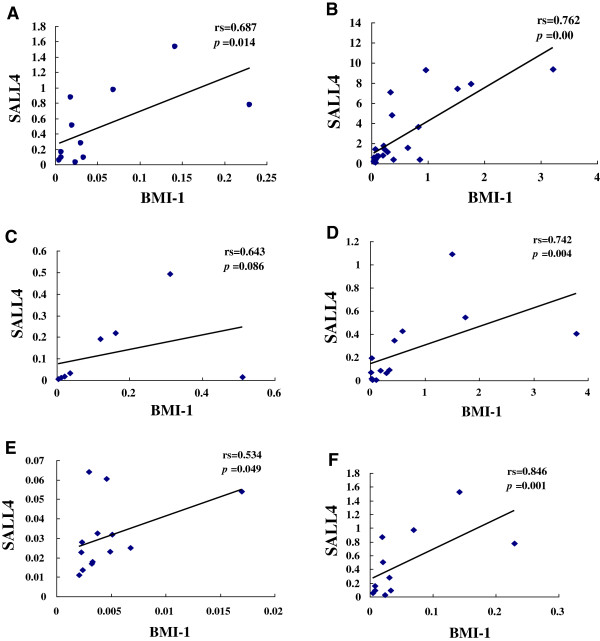
**Correlation analysis of the relative *BMI-*1 and *SALL4 *expression levels in healthy individuals (A), AML (B), AML-CR (C), CML (D), CML-CR (E) and CML-BC (F) groups.** The data of M2, M3, M3-CR, M2-CR, and M5 groups were not shown.

**Figure 4:**
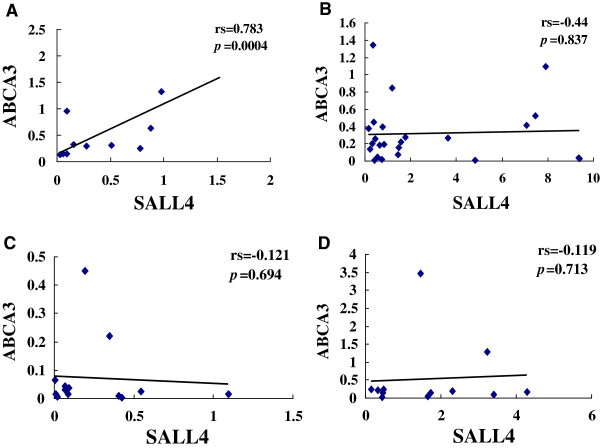
**Correlation analysis of the relative *SALL4 *and *ABCA3 *expression levels in healthy individuals (A), AML (B), CML (C) and the CML-BC (D) groups.**

Correlation analysis of the relative expression levels of *BMI-1* and *SALL4*,*SALL4* and *ABCA3* was performed using Spearman’s rank correlation analysis of the HI, AML and CML groups. A positive expression correlation level for *BMI-1* and *SALL4* genes was found in the HI (rs=0.687, p=0.014), AML (rs=0.762, p< 0.0001), M3 (rs=0.994, p< 0.0001), CML-CP (rs=0.742, p=0.004), CML-BC (rs=1=0.846, p=0.001), M2-CR (rs=1, p < 0.0001) and CML-CR (rs=0.534, p=0.049) groups. However, there was no significant correlation for both genes in the M2 (rs=0.381, p=0.352), M5 (rs=0.643, p=0.086), M3-CR (rs=0.40, p= 0.060) and AML-CR (rs=0.643, p=0.086) groups (Figure 3). A similar result with a positive expression correlation level for genes *SALL4* and *ABCA3* was found in the HI (rs=0.783, p=0.004), while there was no significant correlation between the expression levels of both genes in all myeloid leukemia groups (Figure 4).

## Discussion

*BMI-1* and *SALL4* are stem cell genes that modulate stem cell pluripotency and play a role in leukemogenesis. Dysregulated expression of both genes may have a cooperative effect in leukemogenesis [37]. Patients with RA and RARS who have a higher percentage of BMI-1+ cells showed disease progression to RAEB, suggesting that *BMI-1* is a novel molecular marker that predicts the progression and prognosis of MDS [28]. In this study, we analyzed *BMI-1* and *SALL4* expression in primary AML and CML at diagnosis and those in complete remission.

It has been shown that *BMI-1* overexpression occurs in a variety of cancers including several types of leukemias and lymphomas [38]. In this study, *BMI-1* was found to be overexpressed in AML and chronic phase CML patient groups; and its expression level was lower in patients who achieved complete remission. Similar results were reported by Sawa, M et al. who found that moderate to high *BMI-1* expression was detected in AML patients, and the AML-M0 subtype showed higher relative expression of the *BMI-1* transcript [39]. In addition, Merkerova, M et al. demonstrated that *BMI-1* and its significantly higher *BMI-1* transcript level in CML cells seem to play a secondary role in CML transformation [40]. Our results also indicate that a decreased *BMI-1* expression level is associated with complete disease remission. Interestingly, the *BMI-1* expression level in the CML-BC group appeared to be low in comparison with the de novo CML group, although the difference was not significant. Further investigation is needed using a larger patient cohort to extend our findings. Preliminary results indicate that *BMI-1* may have potential as a therapeutic target for myeloid leukemia. It has been reported that *BMI-1* depletion by RNA interference leads to reduced U937 cell growth and proliferation and increased apoptosis [41], and an antisense *BMI-1* gene can inhibit the growth of K562 cells and upregulate p16 expression in K562 cells [42].

Using immunohistochemistry and real-time PCR, *SALL4* was demonstrated to be constitutively expressed in human primary acute myeloid leukemia [13]. In this study, we found that *SALL4* was overexpressed in different primary AML subtypes, and its expression was lower in the AML-CR patient group. These results are similar to the findings of Jeong, HW et al. who showed that AML patients who responded to treatment had decreasing *SALL4* expression throughout the course of treatment, while AML patients with disease relapse or drug resistance had increasing *SALL4* expression, which was correlated with disease progression [15]. Interestingly, unlike the *SALL4* expression characteristics in AML, the *SALL4* expression level in the CML-CP and CML-CR groups was lower. Moreover, the *SALL4* expression level in patients with chronic phase CML was significantly lower than that in the CML-CR group. There is no direct evidence demonstrating the *SALL4* expression level in CML-CP and comparing the expression feature to healthy individuals; however, Lu and colleagues have found that the SALL4 protein was overexpressed in CML samples in blast crisis but not those in chronic phase by FACS [37]. Our results also demonstrated that *SALL4* expression was higher in the CML-BC group in comparison with the CML-CP and CML-CR groups; however, there was no significant difference in comparison with the HI group. Is it possible that *SALL4* is preferentially expressed in leukemic blasts? These results are similar to a report by Cui W et al. who demonstrated that only precursor B-cell lymphoblastic leukemias/lymphomas and AML had detectable *SALL4* in neoplastic tissues [43]. The different *SALL4* expression patterns in AML and CML suggest that these two disease entities may have different biological characteristics and/or mechanisms of leukemogenesis, at least for the association between *SALL4* and pathogenesis. However, there are not reports comparing the data of SALL4 expression level in CML-CP to healthy individuals, it is difficult to evaluate the significance of this finding. Recently, research from Zhu et al. showed that hematopoietic transcription factor PU.1 expression was significantly lower in newly diagnosed APL patient samples as compared to normal hematopoietic cells, which may relate to the expression level of PML-RARα, and they found that suppression of PU.1 expression occurred concurrently with PML-RARα expression, the authors suggested that low PU.1 expression in APL patients is required for disease initiation and progression [44]. This finding might provide a direction in farther analysis the correlation of *SALL4* with BCR-ABL in the pathogenesis of CML and to address this question.

In principle, the *BMI-1* and *SALL4* gene expression level should be positively correlated in stem cells [9]. Little is known about the expression pattern and differences in the *SALL4* and *BMI-1* genes in patients with AML and CML. In this study, we analyzed the correlation between the relative expression levels of *BMI-1* and *SALL4.* A positive expression level correlation was found for both genes in HI, AML, chronic phase CML, CML-BC and CML-CR patient groups; however, there was no significant correlation between these genes in patients with AML-CR, leaving their role in this group an open question. These results indicate that a positively correlated expression pattern is a common feature in patients with myeloid leukemia and healthy individuals, and both genes may cooperate during cell proliferation and differentiation.

Based on the different expression features of *SALL4* in AML and CML, we further analyzed its regulating gene *ABCA3,* which is a member of the ATP-binding cassette (ABC) family of transport proteins [30,31]. Unlike the description by Wult, Norwood and Steinbach groups, who showed that *ABCA3* is highly expressed in acute meyloid leukemia samples and is associated with unfavorable clinical treatment outcome [24,33,45], in the present study, lower expression level of *ABCA3* was found not only in AML but also in CML groups, especially in CML-CP and CR groups. Moreover, the expression level of *ABCA3* lost the correlation with SALL4 expression in leukemia patients. To determine whether these results relate to favorable clinical outcome, further investigation is needed. Additionally, detection of ABCA2, ABCB2 and ABCC10, which were found overexpressed in childhood AML, may be worthy to build the gene regulation network in proliferation of myeloid leukemia cells.

In conclusion, we determined the expression characteristics of the *SALL4, ABCA3* and *BMI-1* genes in different phases of AML and CML. Further studies will be needed to determine whether *BMI-1* and *SALL4* are novel therapeutic targets for leukemic stem/initiation cells in primary myeloid leukemia.

## Methods

### Samples

Twenty-four newly diagnosed and untreated patients with AML, eight cases with AML in complete remission (AML-CR), thirteen newly diagnosed and untreated patients with CML in chronic phase, 13 cases with CML-CR, and 12 cases with CML in blast crisis (CML-BC),were recruited, the details of the samples was listed in Table 1. The diagnoses of all patients were based on cytomorphology, immunohistochemistry, and cytoimmunological and cytogenetic analysis. Peripheral blood mononuclear cells (PBMCs) from 11 healthy individuals (HI) served as controls. Peripheral blood was collected by heparin anticoagulation, and PBMCs were separated using the Ficoll-Hypaque gradient centrifugation method. All procedures were conducted in accordance with the guidelines of the Medical Ethics committees of the health bureau of Guangdong Province, China.

**Table 1:** The details of samples used in study

**Diagnosis**	**Subtype**	**Numbers**			**Age (year)**	
		**Total**	**Male**	**Female**	**Range**	**Median**
AML		24	12	12	6-69	32
	M2	8	4	4	16-61	42.5
	M3	8	3	5	24-52	30
	M5	8	6	2	6-69	30.5
AML-CR		8	4	4	16-61	32
	M2-CR	4	3	1	39-61	47
	M3-CR	4	1	3	16-25	20
CML-CP		13	11	2	13-64	38
CML-BP		12	6	6	23-66	42.5
CML-CR		13	7	6	15-55	32
HI		11	5	6	24-57	36

### RNA extraction and cDNA synthesis

RNA was extracted using the Trizol kit (Invitrogen, Carlsbad, CA, USA) and then reverse-transcribed into first-strand cDNA using random hexamer primers and the Superscript II reverse transcriptase Kit (Invitrogen) according to the manufacturer’s instructions.

### Real-time quantitative reverse transcription–polymerase chain reaction (qRT–PCR)

The expression levels of *BMI-1*, *SALL4*, *ABCA3* and the *β2-*microglobulin (*β2-MG*) reference gene were determined by SYBR Green I real-time PCR. Briefly, PCR was performed in a 25 μL total volume containing 1 μL of cDNA, 9 μL of 2.5× SYBR Green mix (Tiangen, Beijing, China), and 10 μmol/L primer pairs. After initial denaturation at 95°C for 2 min, 45 cycles consisting of the following procedure were performed using an MJ Research DNA Engine Opticon 2 PCR cycler (BIO-RAD, USA): 15 s at 95°C, 40 s at 64°C for *β2M* and *BMI-1*, 60°C for *SALL4*, and 63°C for *ABCA3*. The relative amounts of the genes of interest and the *β2-MG* reference gene were measured in two independent assays. The data are presented as the relative expression of the genes of interest relative to the internal control gene as determined by the 2^(−∆∆CT)^ method [1-3,5,46]. Additionally, the specific amplification of the PCR products was analyzed by melting curve analysis and agarose gel electrophoresis. The primers used for real-time PCR for all gene amplifications were synthesized by Shanghai Biological Engineering Technology Services Co., Ltd. (Table 2). RT–PCR, for the *BMI-1*, *SALL4* and *ABCA3* genes, was performed using the same primers described above, and the PCR products were sent to Shanghai Invitrogen Biotechnology Co. for DNA sequence analysis.

**Table 2:** Primer sequences used for real-time PCR

**Primer**	**Sequence**	**Accession No**	**PCR product size**
SALL4-f	5’-TGCAGCAGTTGGTGGAGAAC-3’	NM_020436.3	68 bp
SALL4-r	5’-TCGGTGGCAAATGAGACATTC-3’		
BMI-1-f	5’-TAAGCATTGGGCCATAGT-3’	NM_005180.8	140 bp
BMI-1-r	5’-ATTCTTTCCGTTGGTTGA-3’		
ABCA3-f	5’-CTCCGAGAAGGACTTTGAGG-3’	NM_001089.2	144 bp
ABCA3-r	5’-TCCGTGTGTAACTGAACCGT-3’		
β2M-f	5’-TACACTGAATTCACCCCCAC-3’	J00105	144 bp
β2M-r	5’-CATCCAATCCAAATGCGGCA-3’		

### Statistical analyses

Differences in mRNA expression between two groups were analyzed using the Mann–Whitney *U* test. Data are presented as median. Spearman’s rank correlation analysis was used to analyze the *SALL4*, *BMI-1* and *ABCA3* mRNA levels in different samples using the SPSS 11.5 statistical software. Differences were considered statistically significant at *P* < 0.05.

## Competing interests

The authors declare that they have no potential conflicts of interest.

## Authors’ contributions

YQL and YPM contributed to concept development and study design. QS, SCL, SHC and YM performed the real-time PCR. JYH, LJY, BL, XLW, JCY were responsible for collection of clinical data. YQL and QS coordinated the study and helped drafting the manuscript. All authors read and approved the final manuscript.
